# Intralesional injection of mitomycin C following internal urethrotomy of *de novo* bulbar urethral stricture:New experience using a novel adjustable-tip needle

**DOI:** 10.1080/2090598X.2021.1891688

**Published:** 2021-03-01

**Authors:** Yasser A. Noureldin, Abdallah Fathy, Shabib Ahmed, Alaa El Shaer, Saad Ali, Zakaria Saki, Ahmed Sebaey

**Affiliations:** a Department of Urology, Faculty of Medicine, Benha University, Benha, Egypt; b Department of Urology, Milton Keynes University Hospital, Milton Keynes, UK

**Keywords:** Urethral stricture, recurrence, mitomycin C, urethrotomy

## Abstract

**Objectives**: To assess the efficacy of intralesional injection of mitomycin C (MMC), using a novel adjustable-tip needle, following visual internal urethrotomy (VIU) in reducing the recurrence of *de novo* bulbar urethral stricture disease (USD).

**Patients and methods**: Using closed envelopes, 50 patients diagnosed with bulbar USD of <2 cm were randomised to undergo either VIU with MMC injections (Group-A) or VIU alone (Group-B). The urethrotomy was performed under direct vision using a cold-knife and incisions were made at the 12-, 4-, and 8-o’clock positions followed by intralesional injection of 10 mL MMC (0.4 mg/mL) using a novel depth-adjusting needle. All patients were objectively evaluated pre- and postoperatively at 3, 6, and 9 months using uroflowmetry (maximum urinary flow rate), post-void residual urine volume, and retrograde urethrography.

**Results**: Of all 50 patients; five missed follow-up (four in Group-A, one in Group-B), hence 45 cases were available for analysis (21 in Group-A and 24 in Group-B). The mean (SD) age of patients was 34.33 (7.2) and 37.7 (10.2) years in Group-A and Group-B, respectively (*P* = 0.22). The depth-adjusting needle was easy to use and all injections were successfully completed. In terms of stricture recurrence, there was significant decrease in Group-A (three patients, 14.3%) compared with Group-B; (12 patients, 50%) (*P* = 0.01). On multivariate Cox regression analysis, the VIU with MMC was found as a sole factor associated with marked decrease in stricture recurrence (hazard ratio 0.23, 95% confidence interval 0.06–0.93; *P* = 0.04). The Kaplan–Meier survival curve for recurrence-free survival showed a statistically significant difference between both groups (85.7% vs 50.0%; chi-squared = 7.079, *P* = 0.008).

**Conclusion**: The use of a novel depth-adjusting needle was easily applied and MMC injection after VIU resulted in a significantly lower recurrence of *de novo* bulbar USD.

**Abbreviations** : MMC: mitomycin C; PVR: post-voiding residual urine; Q_max_: maximum urinary flow rate; RFS: recurrence-free survival; RUG: retrograde urethrography; USD: urethral stricture disease; VIU: visual internal urethrotomy

## Introduction

Urethral stricture disease (USD) is a term that refers to the scarring process involving the epithelium or spongy erectile tissue of the corpus spongiosum (spongiofibrosis); therefore, it is considered as an anterior urethral disease. USD can be asymptomatic for a while, but because the lumen is further reduced, it can be associated with marked voiding symptoms [1]. There are several procedures for the management of urethral stricture ranging from minimally invasive manoeuvres, e.g. urethral dilatation and visual internal urethrotomy (VIU), to the invasive reconstructive urethroplasty techniques [2].

The VIU is one of the basic methods for the treatment of USD. Being a minimally invasive procedure with less morbidity, it is commonly performed by endourologists around the globe [3]. Success of VIU depends on complete progress of epithelisation before wound contraction. Nevertheless, if wound contraction occurs before completion of the epithelialisation, stricture recurs. Therefore, scientists used anti-fibrotic drugs, such as mitomycin C (MMC), botulinum toxin A, somatostatin analogue and corticosteroids, as an adjunctive therapy to delay wound contraction, hence improving USD recurrence [4,5].

MMC is a chemotherapeutic agent that has potent alkylating anti-neoplastic antibiotic activity by inhibition of DNA synthesis by cross-linking DNA between guanine and adenine, and suppression of cellular RNA and protein production. Hence, it prevents replication of fibroblasts and epithelial cells and inhibits collagen synthesis resulting in delayed wound contraction [6,7]. The MMC injection was previously used for intralesional injection, either before VIU [4] or after VIU [6,8], with encouraging results [4,6,8]. The intralesional injection was performed using traditional cystoscopic needles without the option to adjust the depth, which might have posed some difficulties and bias in the results due to the use of different depths during injection, and the MMC might not have been delivered to the target site. Therefore, the objective of the present study was to assess the efficacy of intralesional injection of MMC, using a novel adjustable-tip needle, following VIU in reducing the recurrence of *de novo* bulbar USD. Our hypothesis was that appropriate intralesional injection of MMC by a special adjustable-tip needle after subjective estimation of the depth of injection, as an adjunctive procedure, can unify the depth of injection along the cohort which undergo injection and make the procedure easier and more successful, the results more reliable, and significantly reduce recurrence of USD after VIU.

## Patients and methods

### Study design

**Figure 1.**
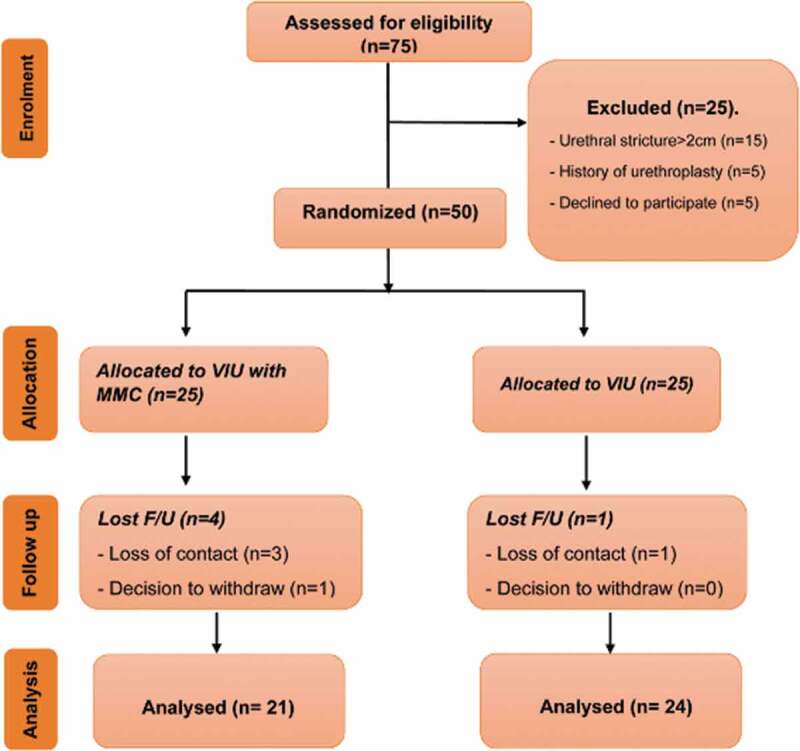
Participants’ flow chart diagram

This randomised controlled study was conducted in our tertiary care urology institute between April 2018 and July 2020 (including the follow-up period), after obtaining ethics approval and informed consents. Randomisation was achieved using closed envelopes. We recruited all patients with single, bulbar USD of a maximum length of 2 cm, with partial thickness spongiofibrosis on sonourethrography and a maximum urinary flow rate (Q_max_) of <12 mL/s. Patients with complex stricture, complicated by a fistula or abscess, blind urethral stricture, history of urethroplasty, and full-thickness spongiofibrosis were excluded. Patients who agreed to participate in this study were randomised into two groups: Group-A (study group) underwent intralesional MMC after VIU and Group-B (control group) underwent VIU only (Figure 1).

All patients were evaluated preoperatively based on full history, clinical examination, routine laboratory investigations, retrograde urethrography (RUG), abdomino-pelvic ultrasound with estimation of post-voiding residual urine (PVR) volume, uroflowmetry to assess Q_max_ and sonourethrography to assess the presence and the depth of spongiofibrosis. Sonourethrography was performed by a professor of diagnostic radiology from our tertiary care centre and the technique was executed as follow: after sterilisation of the external urethral meatus and insertion of a 12-F Foley catheter in the navicular fossa and inflation of its balloon using 2 mL of sterile water for fixing it in place, a continuous injection of normal saline using a 60-mL syringe to gently dilate the urethra and the ultrasound probe (7.5 MHz) is applied to identify the non-distensible narrow area and measure it [9].

### Intervention

**Figure 2.**
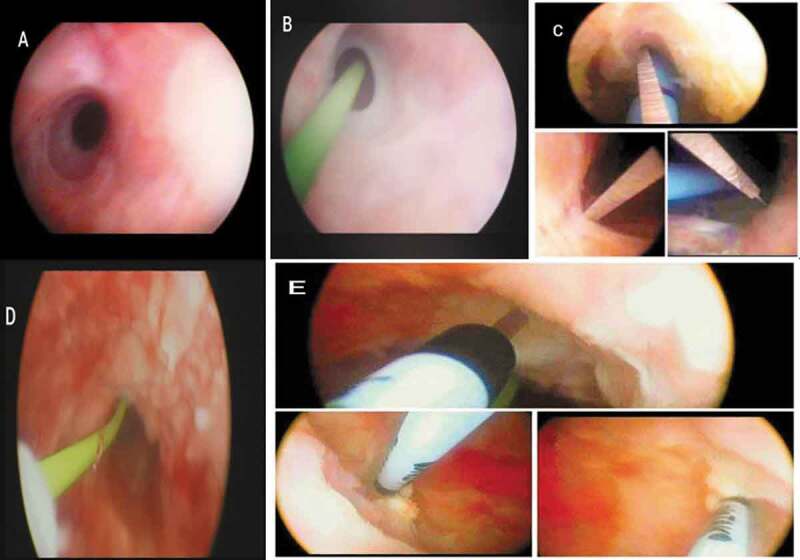
(a) Visualisation of stricture via 17-F rigid cystoscope. (b) Application of a guidewire via the cystoscope. (c) Cold-knife incisions at the 12-, 4- and 8 o’clock positions. (d) Healthier appearing tissue after VIU. (e) Multiple intralesional MMC injections

**Figure 3.**
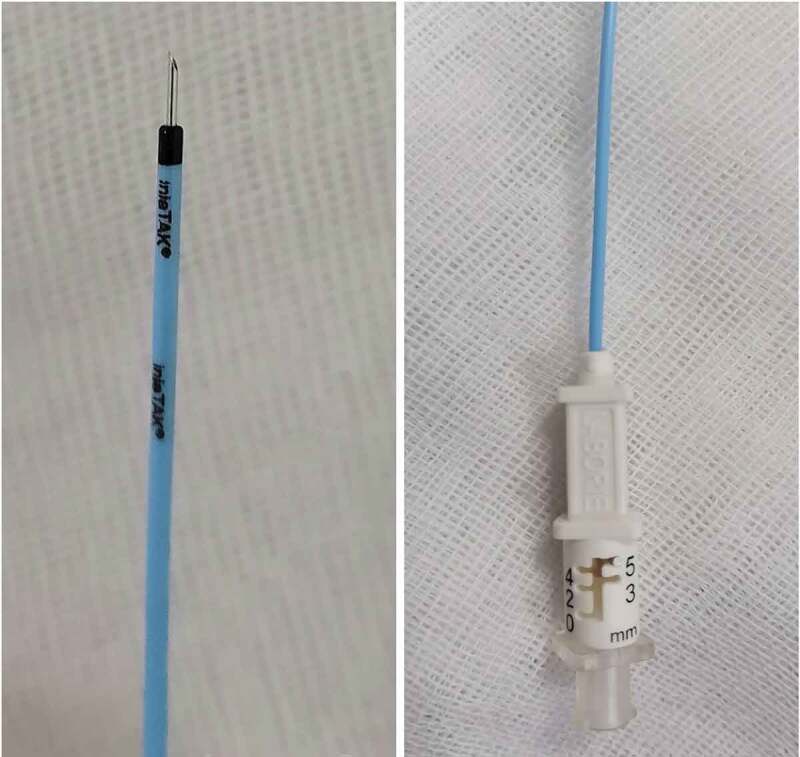
The DIS199: injeTAK® adjustable-tip needle, LABORIE, Williston, VT, USA

The intervention was performed under spinal anaesthesia. Preoperative antibiotic prophylaxis with a single oral dose of levofloxacin 500 mg was given. Patients were put in lithotomy position, and cysto-urethroscopy was performed using a 17-F rigid cystoscope to allow for a guidewire and ureteric catheter to pass through the stricture into the urinary bladder. Cold-knife incisions, at the 12-, 4-, and 8-o’clock positions, were made through the whole thickness of the fibrosis until healthier tissue appeared (Figure 2). In Group-A, a special depth-adjustable injection needle for rigid cystoscope use (DIS199: injeTAK® adjustable-tip needle, LABORIE, Williston, VT, USA) was used to inject 0.4 mg/mL MMC along the whole length of each incision into healthier-appearing tissue (4 mg dose of MMC) (Figure 3). The needle is 35 cm and 23 G/4.8 F.

The depth of injection was subjectively estimated during VIU and the required depth was adjusted (2, 3, 4 or 5 mm) (Figures 2 and Figures 3). The patients in Group-B did not undergo intralesional injections. At the end of procedure, an 18-F silicone urethral catheter was left indwelling for 7 days. The patients were then taken to the recovery room and were discharged the same day with a short course of antibiotics (oral levofloxacin 500 mg, once daily for 5 days).

### Postoperative follow-up

All patients were asked to attend at 1 month for assessment of obstructive symptoms and undergo abdomino-pelvic ultrasonography for calculation of PVR and undergo uroflowmetry for assessment of voiding pattern and calculate Q_max_. All patients were followed-up at 3, 6 and 9 months for clinical assessment of obstructive symptoms, Q_max_, PVR by abdomino-pelvic ultrasonography, and RUG. The main outcome parameter was urethral stricture recurrence, which was identified as recurrence of LUTS during follow-up visits starting at 3 months with difficulty in passing a 16-F urethral catheter and confirmed by RUG [8].

### Statistical analysis

Sample size was calculated using the G*Power 3.1.9.7 for Windows. Based on alpha adjusted at 0.05 and the power adjusted at 80%, and an expected difference in recurrence rate of 40% between Group-A (VIU with MMC injection) and Group-B (VIU only) [4], a total sample size of 40 patients (20 in each group) was required. After allowing for an expected drop out of 20%, the total number of patients required for this study was 50 patients (25 in each group). Data analysis used the Statistical Package for the Social Sciences (SPSS®), version 20.0 (IBM Corp., Armonk, NY, USA). The mean ± standard deviation (SD) was used for presentation of quantitative data, while number (%) was used for qualitative data. The independent samples *t*-test of significance was used when comparing between two means, and the paired sample *t*-test was used to compare the outcomes within groups at 3, 6, and 9 months. The chi-square test of significance was used to compare proportions between two qualitative parameters. A two-tailed *P* ≤ 0.05 was considered significant. The Kaplan–Meier survival curve was used to track recurrence-free survival (RFS). Factors affecting RFS were compared at the univariate level using the log-rank test. Multivariate stepwise Cox regression analysis was used for identification of predictors of recurrence. Hazard ratios (HRs) with 95% CIs were calculated for predictors.

## Results

From a total of 50 randomised patients (25 in each group), five patients (10%) discontinued the study; four of 25 (16%) and one of 25 (4%) patients were lost to follow-up in groups A and B, respectively. Overall, 45 patients were available for analysis; Group-A, 21 patients and Group-B, 24 (Figure 1). All patients in Group-A (study group) received MMC injection at a subjectively estimated depth of 3 mm using the injeTAK adjustable-tip needle.

The mean (SD) age of all patients was 36.13 (9.03) years and the mean (SD) length of the stricture was 1.31 (0.40) cm, the majority of patients (35 patients) had partial thickness spongiofibrosis and only 10 patients had no spongiofibrosis. In terms of the cause of stricture, an idiopathic origin was the most common cause (36 patients), the mean (SD) preoperative Q_max_ was 8.69 (1.55) mL/s and the preoperative PVR was 76 (27.45) mL.

There was no statistically significant difference between groups A and B in terms of patient age (mean [SD] 34.33 [7.23] vs 37.71 [10.26] years; *P*= 0.22), cause of stricture (*P*= 0.71), and length of stricture (mean [SD] 1.24 [0.37] vs 1.42 [0.41] cm; *P*= 0.14; Table 1). According to sonourethrography, most cases in both groups had partial thickness spongiofibrosis, and there was no statistically significant difference between the two groups regarding presence or absence of partial thickness spongiofibrosis (*P*= 0.99; Table 1). Similarly, there was no statistically significant difference between groups A and B in terms of the preoperative Q_max_ (mean [SD] 9.0 [1.73] vs 8.42 [1.35] mL/s; *P*= 0.21) and PVR (mean [SD] 62.71 [25.89] vs 87.62 [23.59] mL; *P*= 0.21; Table 1).

**Table 1.** Baseline perioperative characteristics

Variable	Group-A (*n* = 21)	Group-B (*n* = 24)	*P*
Partial thickness spongiofibrosis, % (*n*)	76.2 (16)	79.2 (19)	0.99
Cause of stricture, % (*n*)			0.71
Idiopathic	76.2 (16)	83.3 (20)	
Inflammatory	23.8 (5)	16.7 (4)	
Length of stricture, cm, mean (SD)	1.24 (0.37)	1.42 (0.41)	0.14
Preoperative assessment of Q_max_, mL/s, mean (SD)	9.0 (1.73)	8.42 (1.35)	0.21
Postoperative assessment of Q_max_, mL/s, mean (SD)			
at 3 months	17.95 (3.38)	4.20 (3.87)	0.001*
at 6 months	17.29 (3.42)	13.76 (3.99)	
at 9 months	17.43 (3.26)	13.63 (3.49)	
*P*_1_	<0.001*	<0.001*	
Preoperative assessment of PVR, mL, mean (SD)	62.71 (25.89)	87.62 (23.59)	0.21
Postoperative assessment of PVR, mL, mean (SD)	29.90 (23.12)	58.75 (38.23)	0.004*
*P*_1_	<0.001*	<0.001*	

Group-A: VIU with MMC injection; Group-B: VIU only (control); *P: P* value comparing between the two studied groups; *P_1_: P* value for paired t-test comparing between pre- and postoperative values.

*Statistically significant at *P*≤ 0.05.

There was no statistically significant difference between the two studied groups for intra- (*P*= 0.99) and postoperative complications (*P*= 0.40); bleeding 9.5% in Group-A (two patients) and 8.3% in Group-B (two), and postoperative fever (only two in Group A and three in Group B). Nevertheless, there was highly statistically significant difference between pre- and postoperative Q_max_ and PVR between the two groups (*P*= 0.001; Table 1). Similarly, there was a statistically significant difference between both groups in the terms of recurrence; three patients in Group-A (14.3%) and 12 in Group-B (50%) had recurrence (*P*= 0.01; Table 2). The RFS on univariate analysis using the log-rank test was associated with age <35 years, intralesional MMC injection, stricture length ≤1 cm, absence of partial spongiofibrosis, and Q_max_ <9 mL/s (Table 3). Nevertheless, on multivariate Cox regression analysis, the VIU with MMC was found as a sole factor associated with marked decrease in stricture recurrence (HR 0.23, 95% CI 0.06–0.93; *P*= 0.04; Table 4).

**Table 2.** Comparison between both groups according to recurrence rate and timing of recurrence

Recurrence, *n* (%)	Group-A (*n*= 21)	Group-B (*n*= 24)	Chi square	*P*
Overall recurrence	3 (14.3)	12 (50.0)	6.429	0.01*
Timing of recurrence				
at 3 months	0 (0)	6 (25)	8.839	0.01*
at 6 months	1 (4.8)	4 (16.7)		
at 9 months	2 (9.5)	2 (8.3)		

Group-A: VIU with MMC injection; Group-B: VIU only (control).

*Statistically significant at *P* ≤ 0.05.

**Table 3.** Univariate analysis for factors affecting RFS

Variable	9-month RFS, %	*P*
Age, years		
<35	81.8	
≥35	52.2	0.029
Group		
A	85.7	
B	50.0	0.008
Cause of stricture		
Idiopathic	63.9	
Inflammatory	77.8	0.435
Length of stricture, cm		
≤1	94.4	
>1	48.1	0.001
Partial thickness spongiofibrosis		
Yes	57.1	
No	100.0	0.017
Q_max_, mL/s		
<9	42.9	
≥9	87.5	0.001
Intraoperative complications		
Yes	65.9	
No	75.0	0.791

**Table 4.** Multivariate Cox regression analysis for predictors of recurrence

Variable	Regression coefficient (B)	HR (95% CI)	*P*
Age ≥35 years	1.039	2.826 (0.83–9.7)	0.09
VIU with MMC	–1.469	0.23 (0.06–0.93)	0.04
Length of stricture ≥1 cm	1.816	6.145 (0.76–49.9)	0.09
Q_max_ ≥9 mL/s	–1.623	0.197 (0.02–1.62)	0.13

**Figure 4.**
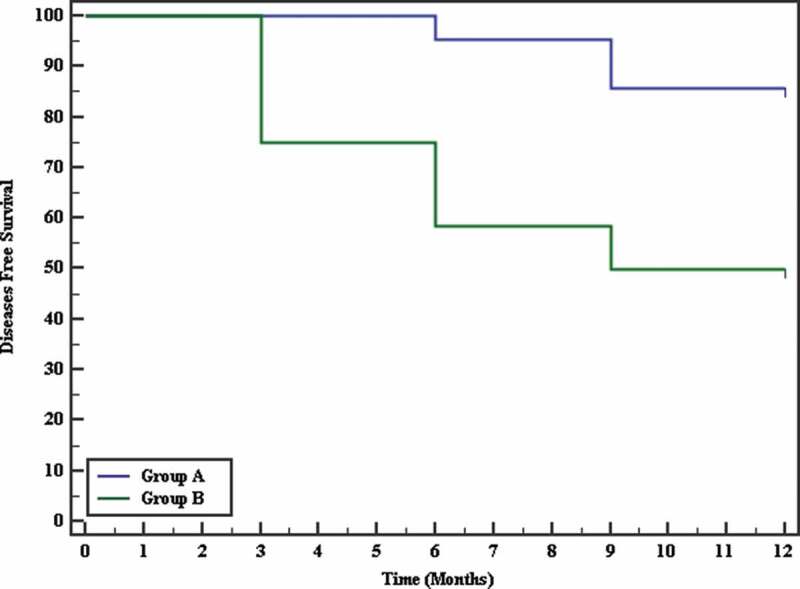
Kaplan–Meier survival curve for diseases-free survival

In the terms of the Kaplan–Meier survival curve for RFS, there was a statistically significant difference between the groups (85.7% vs 50.0%; chi squared = 7.079, *P*= 0.008; Figure 4).

## Discussion

The term of USD is limited to the anterior urethra; other narrow lumens of the posterior urethra are termed urethral contractures or stenoses [10]. The USD is still a common and challenging urological problem. The VIU remains the most commonly performed procedure for treatment of USD, despite the high failure rate, which approaches 40% even when it is initially performed selectively for short bulbar strictures of <2 cm under optimal conditions [11,12]. This has culminated in the expansion of the concept ‘once a stricture, always a stricture’ [13].

In our present study, we used a special adjustable-tip needle (from LABORIE, USA) for injecting MMC, and assessed the effect of this on the recurrence of USD. Of the 50 patients recruited for the present study, four patients in Group-A and one in Group-B did not attend for follow-up in the first 3 months; hence, were dropped from the study. The sample during analysis was thus reduced to 45 patients, Group-A (study group) comprised 21 patients who had intralesional MMC after VIU and Group B (control group) comprised 24 patients who underwent VIU only.

Regarding the demographic assessment of our patients, the mean (SD) age in Group-A was 34.33 (7.23) years, while in Group-B it was 37.71 (10.26) years. This was consistent with the age group of the Ali *et al*. [6] study, in which the mean (SD) age of patients in Group A was 37.31 (10.1) years and in Group B was 40.1 (11.4) years. On the other hand, Mazdak *et al*. [4] operated on patients with a mean (SD) age of 29.8 (14.8) and 29.2 (13.9) years in the MMC-treated and untreated groups, respectively. In the present study, the cause of USD was idiopathic in 76.2% of Group-A patients and 83.3% in Group-B. This differed from the study of Ali *et al*. [6], which reported road traffic injury and iatrogenic urethral injury as the most common causes in both groups. In the present study, the mean (SD) length of USD was 1.24 (0.37) cm in Group-A and 1.42 (0.41) cm in Group-B. This was comparable with the study by Ali *et al*. [6], where the mean (SD) stricture length was 1.86 (1.2) cm in Group A and 1.67 (1.4) cm in Group B. Similarly, Farrell *et al*. [8] operated on a median stricture length of 2 cm. Whereas, the stricture length in this study was longer than the Madzok *et al*. [4] study, where the mean (range) stricture length was 0.76 (0.5–1) mm in Group A and 0.74 (0.5–1) mm in group B.

In the present study, the preoperative mean (SD) Q_max_ was 9.0 (1.73) mL/s in Group-A and 8.42 (1.35) mL/s in Group-B, while the mean (SD) Q_max_ at 9 months was 17.43 (3.26) mL/s in Group-A and 13.63 (3.49) mL/s in Group-B, which was highly statistically significant. These results were congruent with the study of Farrell *et al*. [8], where the Q_max_ on uroflowmetry was significantly increased after VIU with MMC (mean [SD] 9.2 [5.8] vs 15.4 [11.8] mL/s; *P*= 0.02).

In addition to the above results, there was a statistically significant decrease in PVR at 9-months postoperatively compared with the preoperative measurements in both groups A and B, at a mean (SD) of 29.90 (23.12) vs 62.71 (25.89) mL and 58.75 (38.23) vs 87.62 (23.59) mL, respectively. However, the results from the study of Farrell *et al*. [8] found no significant decrease in PVR postoperatively (61.9 vs 92.9 mL; *P*= 0.22). The authors considered this to be due to the relatively low preoperative PVR.

Regarding complications, the present study found that there was no statistically significant difference between the two studied groups for intra- and postoperative complications (bleeding 9.5% in Group-A and 8.3% in Group-B, and only two patients in Group-A and three in Group-B with postoperative fever). There was also no significant difference between the two groups in the study by Ali *et al*. [6].

In terms of stricture recurrence in our present study, there was significant decrease in Group-A (three patients [14.3%], one at 6 months and two at 9 months postoperatively) compared with Group-B (12 patients [50%], six at 3 months, four at 6 months, and two at 9 months postoperatively) (*P*= 0.01). These recurrence rates were comparable with the study by Madzok *et al*. [4], with a 10% recurrence rate in the MMC group compared with 50% in the VIU alone group. However, the lower recurrence in the MMC group (10%) compared with the present study (14%) might have been due to the shorter stricture length in the Madzok *et al*. [4] study where they excluded all strictures >1.5 cm. Ali *et al*. [6] reported a significant reduction in recurrence of stricture to 14.1% in the MMC group compared with 36.9% in the control group. The higher recurrence rate in the non-MMC group in our present study (50%) compared with the Ali *et al*. [6] (36.9%) might be attributed to the difference in the inclusion criteria and the nature of the strictures. On regression analysis, VIU with MMC was found to be the sole factor associated with the marked decrease in stricture recurrence (HR 0.23, 95% CI 0.06–0.93; *P*= 0.04).

Limitations of the present study include the relatively short period of follow-up. However, USD recurrence usually occurs in the first 3–6 months. Another limitation is that all patients were followed-up by RUG because we do not have flexible ureteroscopy on site. Also, patients without spongiofibrosis on sonourethrography were not randomised between the two groups. Additionally, we did not plan to assess the safety of MMC injection following VIU. However, no adverse events related to MMC injection were identified during the follow-up period. Nevertheless, the present study is the first to use a novel depth-adjusting needle for intralesional injection of MMC after VIU and this study adds to the scant literature about the efficacy of intralesional MMC injection following VIU of bulbar USD.

## Conclusion

The use of a novel depth-adjusting needle was easily applied for MMC injection after VIU and MMC injection after VIU resulted in a significantly lower recurrence of *de novo* bulbar USD. Other multicentre prospective studies recruiting more patients are encouraged.
